# Identification of Aly1 and Aly2 as Modulators of Cytoplasmic pH in *Saccharomyces cerevisiae*

**DOI:** 10.3390/cimb46010013

**Published:** 2023-12-25

**Authors:** Guoyong Liu, Xiuli Han, Xiang Yu, Yu Wang, Jinbiao Ma, Yongqing Yang

**Affiliations:** State Key Laboratory of Plant Environmental Resilience, China Agricultural University, Beijing 100193, China; gyl211307@163.com (G.L.); hanhxl@sdut.edu.cn (X.H.); judge_b@126.com (X.Y.); yw2250@cau.edu.cn (Y.W.);

**Keywords:** *Saccharomyces cerevisiae*, plasma membrane H^+^-ATPase, *PMA1*, suppressor, *Aly1/2*

## Abstract

The regulation of intracellular pH in yeast (*Saccharomyces cerevisiae*) cells is critical for cell function and viability. In yeast, protons (H^+^) can be excreted from the cell by plasma membrane ATPase PMA1 and pumped into vacuoles by vacuolar H^+^-ATPase. Because PMA1 is critical to the survival of yeast cells, it is unknown whether other compensatory components are involved in pH homeostasis in the absence of PMA1. To elucidate how intracellular pH is regulated independently of PMA1, we employed a screening approach by exposing the yeast haploid deletion mutant library (ver 4.0) to the selective plant plasma membrane H^+^-ATPase inhibitor PS-1, which we previously reported. After repeated screenings and verification, we identified two proteins, *Aly1* and *Aly2*, that play a role in the regulation of intracellular pH when PMA1 is deficient. Our research uncovers a new perspective on the regulation of intracellular pH related to PMA1 and also preliminarily reveals a role for *Aly1* and *Aly2* in the regulation of intracellular pH.

## 1. Introduction

Proper cytosolic pH homeostasis is crucial for yeast survival since protons (H^+^) in the cytosol act not only as facilitators for a series of cellular activities but also as potent second messengers in a variety of physiological programs, such as growth, development, and stress responses [1]. pH homeostasis is tightly related to nutrient signaling, and nutrient supply can regulate cytosolic pH homeostasis [2]. For instance, carbon source can induce a time-course intracellular pH change when glucose is added to the cultures of yeast cells. An intracellular acidification was observed due to glucose glycolysis, and then an intracellular alkalinization was observed due to the activity of plasma membrane H^+^-ATPase [3]. Phosphate metabolism in yeast cells is interconnected with intracellular pH since the uptake of inorganic phosphate, its subsequent intracellular distribution, and its conversion into polyphosphates (PolyP) are dependent on intracellular pH homeostasis; moreover, polyphosphate metabolism contributes to the regulation of cytosolic pH [4]. In addition to functions in nutrient signaling, cytosolic pH homeostasis also plays a role in protein trafficking along the yeast secretory pathway: aberrant cytosolic pH can lead to the mislocalization of plasma membrane proteins, such as PMA1 [5].

PMA1 localized to the plasma membrane is the primary determinant of cytosolic pH by pumping cytoplasmic H^+^ out of the cell while also creating the necessary electrochemical gradient for H^+^-coupled nutrient solute uptake, making PMA1 an essential component of multiple cellular physiological activities in yeast cells [6]. Glucose stimulates PMA1 activity and is a carbon source for yeast growth [7]. Budding yeast grown in glucose-limited conditions displays decreased PMA1 activity and depolarized membrane potential in an acidic cytosolic environment, suggesting that PMA1 has an essential role in the coordination of cellular pH and metabolism [2,8]. PMA1-mediated regulation of cytosolic pH also generates a potential second messenger of glucose availability in the form of H^+^ to conserve energy in the face of environmental stress [2]. In response to glucose supply, the C-terminal tail of PMA1 is a major site of regulation via phosphorylation on Ser/Thr residues, thereby alleviating the autoinhibition imposed by this region on PMA1 activity in glucose-starved yeast cells; the identity of the protein kinases that phosphorylate PMA1 is still not clear [2,9]. PMA1 is also a key element involved in promoting the target of rapamycin complex 1 (TORC1) activation in response to H^+^-coupled nutrient uptake [10] and plays an essential role in PolyP metabolism [11]. In addition to nutrient signaling, PMA1 in budding yeast is also involved in various stress responses, such as exposure to weak acids, reactive oxygen species production, and ethanol treatment [12]. Studies of the replicative lifespan of budding yeast showed that endocytic genes such as vacuolar protein sorting 8 (*Vps8*), *Vps9*, and *Vps21* are important for PMA1 accumulation in mother cells during cell division, although PMA1 accumulation in mother cells does not reflect the age of mother cells [13]. As PMA1 function is essential for yeast growth, pharmacological drugs have been designed for use in humans using PMA1 as an antifungal target [14].

Vacuolar H^+^-ATPase (V-ATPase) usually acts in close coordination with PMA1 to regulate cellular pH [1]. V-ATPase is a highly conserved H^+^ pump that translocates H^+^ from the cytosol to the vacuolar compartment, thereby regulating the luminal acidification of multiple biosynthetic and endocytic organelles in various cellular responses, such as vesicle trafficking and pH homeostasis [15]. V-ATPase acts interdependently with PMA1 in pH regulation and yeast growth [16,17,18]. Upon addition of glucose to glucose-deprived yeast cells, the transient cytosolic acidification and a subsequent alkanization of cytosolic pH is delayed in the mutant strains vacuolar membrane atpase3 (*vma3*Δ) and vacuolar pH1 (*vph1*Δ) lacking vacuolar H^+^-ATPase activity, which are defective in the regulation of vacuolar pH [19]. Ubiquitin ligase reverses Spt-phenotype5 (Rsp5), and the arrestin-related adaptor protein regulator of IME2 8 (Rim8) mediates the ubiquitination, internalization, and degradation of Pma1 in *vma*Δ mutants to balance overall pH [17]. The cross-talk between V-ATPase and PMA1 offered a functional basis for exploring the compensatory downregulation of PMA1 in V-ATPase mutant strains, resulting in the isolation of the two phosphatases calcium-responsive calcineurin and glucose-sensitive phosphatase glycogen 7 (Glc7), which is essential for ubiquitination and endocytic downregulation of PMA1 when V-ATPase activity is compromised [18].

Although the compensatory ubiquitination, internalization, and degradation of PMA1 have been uncovered and largely studied in yeast cells when V-ATPase is defective, whether a similar compensatory mechanism exists in yeast cells lacking PMA1 activity is unknown. Here, we report on the identification of the two arrestin genes *arrestin-like yeast protein1* (*Aly1*) and *Aly2* based on their negative synthetic genetic interactions with PMA1 deficient mutants. We demonstrate that Aly1 and Aly2 serve as modulators of cytoplasmic pH when PMA1 is deficient in yeast.

## 2. Materials and Methods

### 2.1. Yeast Strains

The library of haploid deletion mutants (ver 4.0) (purchased from BIONEER company, Taejon, South Korea (visit: https://www.bioneer.co.kr/catalogsearch/result/?q=+pombe+deletion) accessed on 21 December 2023) and SPJ1929 (h+, leu1-32, ura4-D18, ade6-216) strains are in fission yeast (*Schizosaccharomyces pombe*). The AH109 (MATa, *trp1–901*, *leu2–3*, *112*, *ura3–52*, *his3–200*, *gal4Δ*, *gal80Δ*, LYS2::GAL1_UAS_-GAL1_TATA_-HIS3, GAL2_UAS_-GAL2_TATA_-ADE2, and URA3:: MEL1_UAS_-MEL1_TATA_-*lacZ*) and RS-72 (MATa, ade1-100, his4-519, leu2-3, 112, and ura3-52) strains are *Saccharomyces cerevisiae* strains. The AH109 strain is widely used in the yeast two-hybrid system (Y2H) for detecting interacting proteins. The AH109 strain is *gal4*^−^ and *gal80*^−^; this prevents interference of native regulatory proteins with the regulatory elements in the two-hybrid system. AH109 features three reporters, *ADE2*, *HIS3*, and *MEL1* (or *lacZ*), under the control of distinct GAL4 upstream activating sequences (UASs) and TATA boxes. In yeast strain RS-72, *GLCpro:PMA1* was replaced with *GALpro:PMA1* in the genome thus the gene encoding endogenous yeast H^+^ pump, *PMA1*, was under the control of the *GAL1* promoter, making yeast viable only when grown on a medium with galactose as a carbon source, as previously reported [20]. Strains were grown in rich medium (YPD) containing 1% (*w*/*v*) yeast extract, 2% (*w*/*v*) peptone, and 2% (*w*/*v*) glucose (or 2% [*w*/*v*] galactose where specifically indicated).

### 2.2. Gene Deletion Assay

The deletion sequences of the genes in the yeast strains RS-72 were replaced by the coding sequence of the hygromycin resistance gene (*HygR*). A homologous recombination method was used to generate the deletion mutants. In brief, a PCR fragment was generated by the primers of 5′-aly1-HygR-F and 3′-aly1-HygR-R (Table S1) using the vector of pAG32 as the template. Then, recombination was performed. The positive clone of *aly1*Δ was confirmed by the primers of check-aly1-F and check-HygR-R. The primers used to generate other mutants and confirmation primers are listed in Table S1.

### 2.3. Suppressor Screening Assay

In the first screening, there were 95 mutant strains and 1 control strain on one plate that were dipped with a toothpick; each strain was spotted four times, for a total of 36 plates to screen the entire collection. In the second screening, each mutant strain and 1 control strain were dipped on the medium eight times. In the third screening, each strain was spotted with serial dilutions from liquid cultures onto the medium.

### 2.4. SNARF-AM Staining Assay

Yeast cells were collected by centrifugation and washed in ddH_2_O twice before being incubated with 10 μM SNARF-AM for 1 h. The yeast cells were incubated with 0.2% (*v*/*v*) Triton X100 and 10 μM SNARF-AM in a buffer of the indicated pH. The fluorescent signals were visualized with a Nikon CSU W1 confocal microscope (Nikon Corporation, Tokyo, Japan) at 488 nm excitation with 580 nm and 640 nm emission individually. The SNARF-AM dye was purchased from Thermo Fisher, Waltham, MA, USA; more details can be obtained at https://www.thermofisher.cn/cn/zh/home.html accessed on 21 December 2023.

## 3. Results

### 3.1. Screening for New Components Possibly Involved in Cytosolic pH Homeostasis

**Figure 1:**
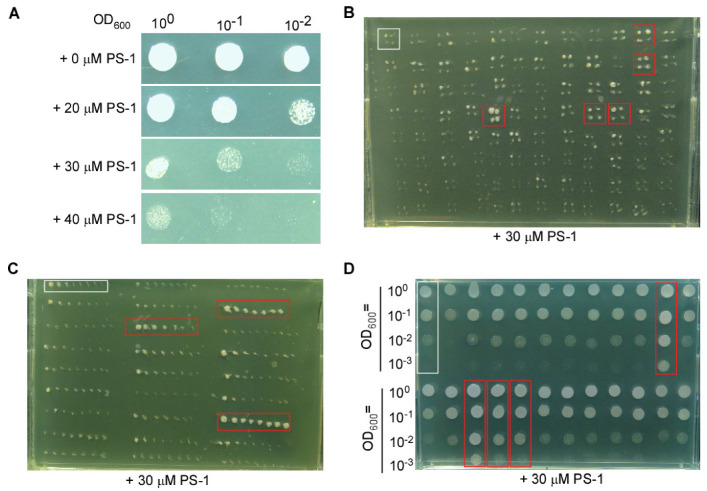
Screening for suppressors that can grow on medium containing PS-1. (**A**) Growth of the fission yeast strain SPJ1929 is inhibited by PS-1. (**B**) Representative photograph of a plate from the first screen for suppressors with better growth than the control on medium containing PS-1. (**C**) Representative photograph of a plate from the second screen for suppressors. (**D**) Representative photograph of a plate from the third screen for suppressors. The control were indicated in the white boxes, the suppressors were indicated in the red boxes.

Multiple physiological activities in yeast cells rely on the maintenance of cytosolic pH, in which PMA1 plays a major role by excreting H^+^ out of the cell. To identify components that might compensate for the loss of PMA1 function in cytosolic pH homeostasis, we looked for mutants that can sustain growth in the absence of PMA1 activity. We previously identified PS-1 as a small molecule inhibitor of plant plasma membrane (PM) H^+^-ATPase [21]. PS-1 might inhibit yeast PMA1, as the heterogeneous expression of a plant PM H^+^-ATPase gene in yeast cells was able to rescue the growth of a yeast *pma1* mutant [21]. As shown in Figure 1A, the control yeast strain SPJ1929 showed vigorous growth in a glucose-containing medium; however, the addition of PS-1 to the medium gradually suppressed the growth of yeast cells in a dose-dependent manner, with 30 μM PS-1 having a substantial negative effect on yeast growth. This result indicates that the activity of yeast PMA1 can also be inhibited by PS-1, thus offering a means to screen for additional components involved in pH homeostasis in the absence of PMA1.

We performed genome-wide rescue screening on a collection of single-gene deletion yeast strains in the *S. pombe* haploid deletion mutant set ver 4.0 (M-4030H), which consists of 3400 mutants. Specifically, we looked for mutants that could still grow when 30 μM PS-1 were added to the culture medium. We carried out the first screening by spotting 95 mutant strains and 1 control strain on one plate with a toothpick, each strain being spotted four times, for a total of 36 plates to screen the entire collection. In the first screening (Figure 1B), the growth of most yeast mutants was suppressed by PS-1 to the same extent as the control strain SPJ1929 (indicated in the white square). However, some mutants appeared to show better growth than the control strain, suggesting that negative components might exist to regulate pH in the absence of PMA1 activity.

From the first screening, we obtained 115 mutants with some degree of rescue compared to the control strain SPJ1929 on medium containing 30 µM PS-1. To reduce the errors and narrow the screen range, we rescreened all 115 mutants in a second screening, with 26 mutants screened simultaneously on one plate as eight spots from a toothpick. Some mutant strains showed better growth compared to the control strain SPJ1929 (indicated in the white square), although most mutant strains were comparable to the control strain, reducing the number of candidates to 33 mutants (Figure 1C). To quantitatively compare the growth rate of the yeast cells, we performed a third screening by spotting serial dilutions from liquid cultures onto medium containing 30 µM PS-1, yielding 24 mutant strains with reproducible better growth than the control strain (Figure 1D). PCR analysis of each mutant strain revealed that 3 mutant strains did not carry a deletion in their intended target gene, while the remaining 21 mutants did.

### 3.2. The Loss of Aly1 and Aly2 Function Can Rescue Growth of Yeast Cells with Reduced PMA1 Activity

PMA1 is essential for yeast survival. To further verify the function of these screened genes in the regulation of pH homeostasis is independent of PMA1, we tested the function of these 21 genes in the budding yeast strain RS-72, which can grow on galactose-containing medium but not on glucose-containing medium because expression of the endogenous *PMA1* gene is placed under the control of the galactose-inducible *galactokinase1* (*GAL1*) promoter (*Pro_GAL_*:*PMA1*), providing an efficient means to selectively shut down *PMA1* expression [20,22]. As the initial mutant screening was performed in *Schizosaccharomyces pombe* (fission yeast), we searched the RS-72 genome for genes homologous to the 21 candidate genes. We detected putative orthologs for 16 genes in *Saccharomyces cerevisiae* (budding yeast) (Table 1); the remaining 5 genes from fission yeast seemed to lack orthologs in budding yeast.

**Figure 2:**
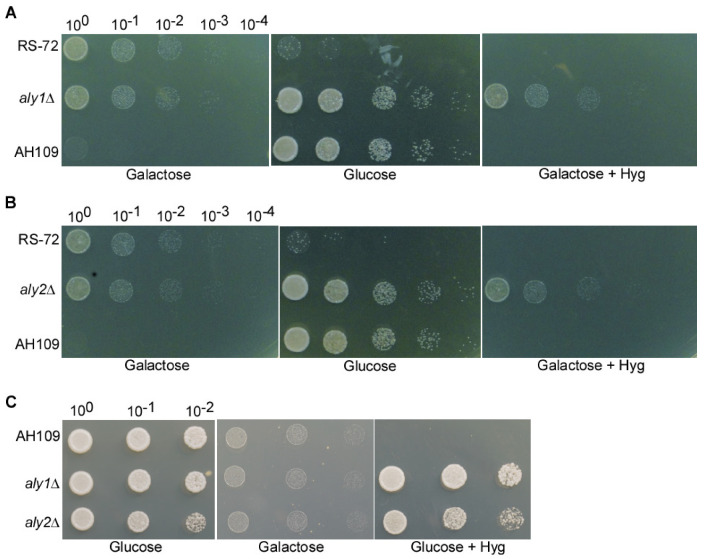
The loss of Aly1 or Aly2 function can rescue yeast growth in *PMA1*-deficient strains. (**A**) Deletion of *Aly1* in the budding yeast strain RS-72 can rescue growth when a reduction in *PMA1* expression due to growth on the medium with glucose is the sole carbon source. (**B**) Deletion of *Aly2* in the budding yeast strain RS-72 can rescue growth when a reduction in *PMA1* expression due to growth on the medium with glucose is the sole carbon source. (**C**) Deletion of *Aly1* or *Aly2* in the budding yeast strain AH109 strain cannot rescue yeast growth on medium with galactose as the sole carbon source. Galactose + Hyg: the control to illustrate that *Aly1* or *Aly2* were replaced with the hygromycin gene using homologous recombination in the RS-72 background. Glucose + Hyg: the control to illustrate that *Aly1* or *Aly2* were replaced with the hygromycin gene using homologous recombination in the AH109 background.

We deleted each of these 16 genes in the RS-72 background and assessed the resulting growth phenotypes when grown on glucose- or galactose-containing medium (Figure S1). We determined that one mutant with a deletion in *Aly1* (*aly1*Δ) could grow well both on glucose- and galactose-containing media, in contrast to RS-72, which only grew on medium containing galactose. By contrast, as a control which can only use glucose as a carbon source, the *Saccharomyces cerevisiae* strain AH109 only grew on glucose-containing medium (Figure 2A). This result suggests that the loss of Aly1 function can rescue yeast growth when PMA1 activity is diminished.

We also assessed the contribution of the Aly1 paralog Aly2 to pH regulation by deleting the gene in the RS-72 background. The *aly2*Δ yeast strain showed the same phenotype as the *aly1*Δ deletion strain, growing both on glucose- and galactose-containing media (Figure 2B). We concluded that Aly1 and Aly2 participate in the regulation of yeast growth in a non-redundant manner.

We also individually deleted Aly1 or Aly2 in the yeast strain AH109 to investigate whether carbon source use was disturbed in *aly1*Δ and *aly2*Δ mutants. The *aly1*Δ and *aly2*Δ mutants grew on glucose-containing medium but not on galactose-containing medium (Figure 2C). Additionally, the growth of AH109, *aly1*Δ, and *aly2*Δ can also be inhibited by PS-1, and *aly1*Δ and *aly2*Δ showed better growth than the AH109 strain (Figure S2). This result suggests that the loss of Aly1 or Aly2 function does not disturb the use of carbon source, indicating that the rescued growth seen for *aly1*Δ and *aly2*Δ on glucose-containing medium in the RS-72 background was due to decreased PMA1 activity.

### 3.3. The Loss of Aly1 or Aly2 Function Promotes H^+^ Extrusion in Yeast Cells with Reduced PMA1 Activity

**Figure 3:**
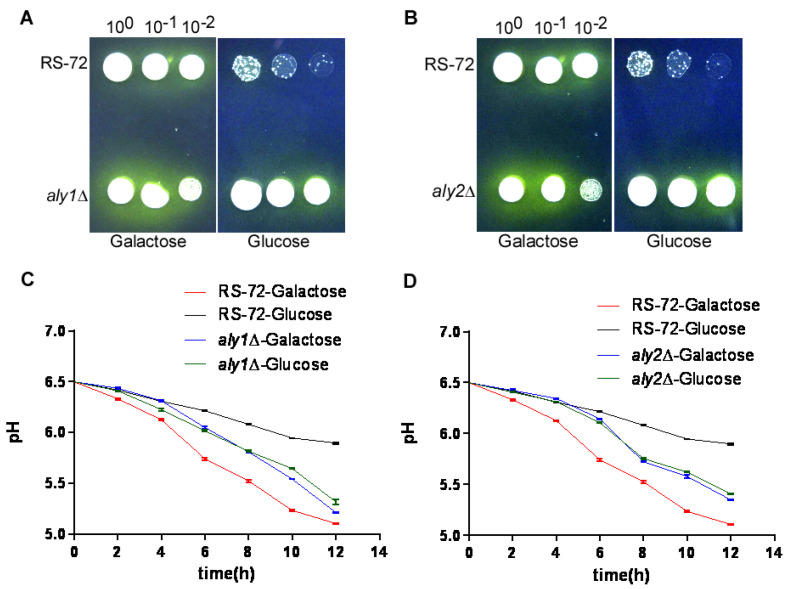
The loss of Aly1 or Aly2 function promotes H^+^ extrusion in yeast cells with reduced PMA1 activity. (**A**,**B**) Visualization of the acidification of growth medium using the pH-sensitive dye bromocresol purple. (**C**,**D**) Acidification of the yeast growth medium over time in *aly1*Δ (**C**) or *aly2*Δ (**D**) deletion strains. Values were taken from 3 independent repeated experiments. Error bars, means ± SD.

We looked for evidence of H^+^ extrusion in yeast cells using the pH indicator bromocresol purple, which changes color from purple to yellow upon acidification. We added the pH indicator to galactose- or glucose-containing medium, which turned purple at the beginning of the growth period (Figure 3A,B). When provided galactose, RS-72, *aly1*Δ, and *aly2*Δ spots acquired yellow halos around them, indicative of medium acidification and thus H^+^ extrusion. By contrast, RS-72 cells failed to grow on glucose-containing medium, whereas both *aly1*Δ and *aly2*Δ mutants displayed strong growth and H^+^ extrusion, as evidenced by the yellow color of the growth medium. This result suggests that the loss of Aly1 or Aly2 may be helpful in alleviating the repression of other components participating in H^+^ extrusion in addition to PMA1.

As a more quantitative method, we measured growth medium pH over time in RS-72 and the *aly1*Δ and *aly2*Δ mutants grown in the presence of glucose or galactose. The pH of RS-72 cultures dropped by approximately 1.5 pH units within 12 h when grown in galactose-containing medium, with a more modest decrease of 0.6 pH units when grown in glucose-containing medium (Figure 3C,D). In contrast to RS-72, *aly1*Δ and *aly2*Δ cultures showed a comparable rate of medium acidification of 1–1.3 pH units regardless of carbon source, indicating that the loss of Aly1 and Aly2 function can promote H^+^ extrusion even in the absence of functional PMA1. The slower acidification rate of *aly1*Δ and *aly2*Δ mutants compared with RS-72 in galactose-containing medium might reflect their slower growth rate. These results suggest that PMA1-mediated H^+^ extrusion is essential for yeast growth; Aly1 and Aly2 play a negative role in H^+^ extrusion in PMA1-defective yeast strains, and the loss of Aly1 or Aly2 function can rescue H^+^ exclusion to promote yeast growth.

### 3.4. The Loss of Aly1 or Aly2 Function Recovers Cytosolic pH in Yeast Cells with Reduced PMA1 Activity

**Figure 4:**
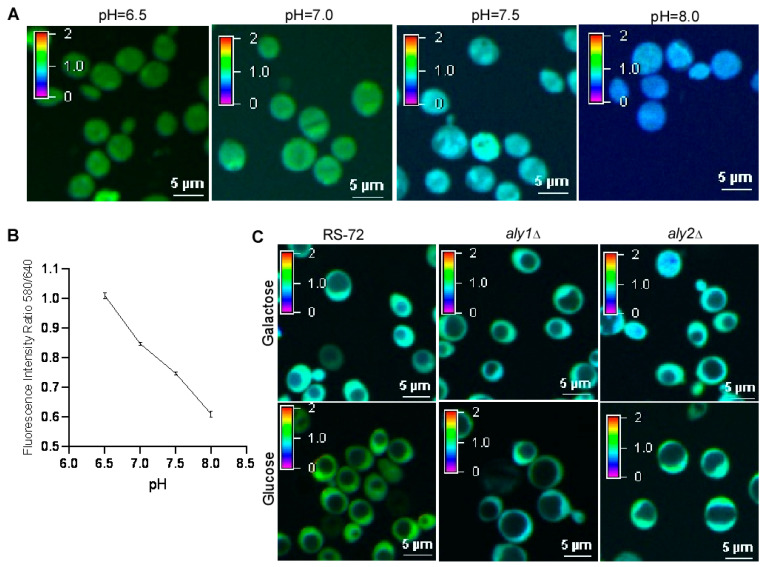
The loss of Aly1 or Aly2 function recovers cytosolic pH in yeast cells with reduced PMA1 activity. (**A**) Representative confocal images of yeast cells stained with SNARF-AM in a buffer containing Triton X100 with different pH values using strain AH109. Yeast cells were shake-cultured in glucose-containing medium with pH values as shown in the figure, and the cells were collected by centrifugation, washed with ddH_2_O, stained with 10 μM SNARF-AM with 0.2% (*v*/*v*) Triton X100, and images were captured by the confocal microscope at 488 nm excitation with 580 nm and 640 nm emission individually. (**B**) Standard curve of fluorescence intensity ratio of 580 nm/640 nm as a function of pH for the cells shown in (**A**), values were taken from 3 independent repeated experiments. Error bars, means ± SD. (**C**) Representative confocal images indicating the pH value in the cytosol of RS-72, *aly1*Δ, and *aly2*Δ yeast cells. The yeast cells were collected by centrifugation, washed with ddH_2_O, stained with 10 μM SNARF-AM with 0.2% (*v*/*v*) Triton X100, and images were captured by the confocal microscope at 488 nm excitation with 580 nm and 640 nm emission individually.

Although the loss of Aly1 and Aly2 function promotes H^+^ extrusion when PMA1 is absent, whether cytosolic pH changes in these mutants is unknown. We used the fluorescent-based cytosolic pH dye SNARF-AM to evaluate changes in intracellular pH by measuring the fluorescence intensity ratio between 580 nm and 640 nm in the cytosol. When indicated by a pseudocolor scale, a more acidified environment in the cell produces a greener color, while a more alkaline environment produces a bluer color (Figure 4A). The fluorescence intensity ratio of 580 nm/640 nm showed a linear relationship with cytosolic pH (Figure 4B).

We thus incubated RS-72, *aly1*Δ, and *aly2*Δ yeast cells grown in galactose-containing medium or glucose-containing medium with SNARF-AM. We established that cytosolic pH in RS-72 cells was lower when grown in glucose-containing medium than in galactose-containing medium. This result indicates an acidified environment when *PMA1* is expressed and supports the idea that PMA1 mediates H^+^ extrusion during yeast growth. By contrast, *aly1*Δ and *aly2*Δ cells showed a more alkaline pH when grown in either glucose-containing medium or galactose-containing medium. This result also supports the idea that the regulation of cytosolic pH consists of other components besides PMA1, with Aly1 and Aly2 acting as negative regulators of cytosolic pH in the absence of PMA1.

## 4. Discussion

Aly1 and Aly2 belong to the α-arrestin family of proteins, which comprises 14 members in budding yeast. This family serve as trafficking adaptor proteins in the regulation of signal-induced PM protein endocytosis and intracellular sorting of nutrient permeases [23,24]. α-arrestins are also named arrestin-related trafficking adaptors (ARTs); Aly1 is called Art6, and Aly2 is called Art3. ARTs possess PY motifs that can interact with the WW domain of ubiquitin ligase Rsp5 to form various ART–Rsp5 complexes [25,26]. ART–Rsp5 complexes play key roles in the regulation of the composition of PM proteins through endocytosis and recycling. For example, resistance to the O-dinitrobenzene 1 (Rod1)–Rsp5 complex regulates endocytosis and recycling of Jen1 (a yeast monocarboxylate transporter) in response to glucose addition and glucose removal, respectively [27]. The regulation of the ART–Rsp5 is complex and coordinated, as is that of the endocytosis of amino acid transporters (AATs), which is activated by the Art2–Rsp5 complex through interaction between the basic patch of Art2 and the C-terminal acidic sorting motifs of AATs during amino acid starvation and also activated by the Art1–Rsp5 complex through the interaction of Art1 and the N-terminal acidic sorting motifs of the same AATs under amino acid-replete conditions [28].

Aly2 localizes to both the PM and the internal compartments of endosomes and *trans*-Golgi network (TGN), and its paralog Aly1 localizes to the internal compartments. Aly1 and Aly2 regulate PM endocytosis and intracellular trafficking in response to various cues. Both proteins can bind directly to the y-subunit of clathrin adaptors AP-1 and Apl4, and regulate the intracellular sorting of Gap1, a general amino acid permease, thus mediating Gap1 trafficking from the TGN to the endosome/vacuole under nitrogen-replete conditions and Gap1 recycling from endosomal compartments back to the TGN and/or the PM to under nitrogen-starvation conditions [26]. Aly1 and Aly2 mediate the endocytosis of Dip5 (dicarboxylic amino acid permease 5), an aspartic acid/glutamic acid transporter [23]. The dephosphorylation of Aly1 by calcineurin is required for the internalization of Dip5 from the PM to the vacuole but not for the intracellular sorting of Gap1 [24]. In addition to nutrient transporters, Aly2 is also involved in the endocytosis of Acr3, an arsenite and antimonite transporter, to promote Acr3 proteolysis in the vacuole [29]. Aly1 and Aly2 can also promote K^+^, inwardly rectifying channels in mammal (Kir2.1) trafficking to the PM, and enhance intracellular potassium levels in a yeast model [30]. In our study, the loss of Aly1 or Aly2 function caused a rise in intracellular pH and rescued yeast growth when PMA1 was deficient. The protein composition at the PM is tightly regulated in response to various physiological environments to maintain intracellular homeostasis of ions, nutrients, and pH. Although the regulatory role of Aly1 and Aly2 was partially uncovered here, their detailed function and mechanism in pH homeostasis regulation is still unknown. Whether the increased endocytosis of nutrient transporters from the PM or the decreased recycling of these nutrient transporters back to the PM is affected by the loss of Aly1 and Aly2 requires further investigation.

Lower intracellular pH can regulate phospholipid metabolism when yeast cells are cultured in glucose-starvation conditions, which is accomplished by the binding of a pH biosensor, phosphatidic acid (PA), to an overproducer of inositol 1 (Opi1) to further repress phospholipid metabolic genes [31]. In addition to intracellular pH, Aly1 and Aly2 may also participate in the regulation of lipid metabolism. Aly1 and Aly2 regulate the trafficking of glycerophosphoinositol 1 (Git1), a transporter for glycerophosphoinositol (GPI), and the loss of Aly1 and Aly2 function might influence the phosphoinositol (PI) balance, e.g., by increasing phosphatidylinositol-3-phosphate (PI3P) on the limiting membrane of the vacuole [32]. A change in the composition of membrane lipids might influence the trafficking of membrane proteins. Some nutrient proteins are clustered at the PM in sphingolipid- and ergosterol-rich membrane compartments occupied by Can1 (MCCs) and respond to their substrates by conformational changes and lateral movement from the MCCs to surrounding lipid domains, leading to their endocytosis [25]. How the composition of lipids might have changed following the loss of Aly1 or Aly2 function in the absence of PMA1, and whether the loss of Aly1 or Aly2 function rescues intracellular pH homeostasis through regulating the lipid balance, requires further study.

Since PMA1 and V-ATPase act interdependently in the regulation of intracellular pH, V-ATPase is thought to be regulated to balance intracellular pH. When V-ATPase is deficient, PMA1 is degraded through the Rim8–Rsp5 complex to balance intracellular pH [17]. However, whether and how vacuolar H^+^-ATPase is regulated to balance intracellular pH when PMA1 is defective has not been reported yet. PMA1 and V-ATPase act coordinately in response to various cues, such as glucose-replete or deprivation conditions. When glucose supply is sufficient, intracellular pH decreases, which leads to the activation of PMA1 to pump protons out of the cell but also leads to the activation of V-ATPase through the assembly of the V1 and Vo subunits to pump protons into the vacuole [6]. However, when glucose is depleted, PMA1 becomes inactive, and the V1 and Vo subunits of V-ATPase separate to decrease V-ATPase activity [6]. The activation of V-ATPase is dependent on external pH, as rapid vacuole acidification can be observed at external pH 5.0 while alkalinization at external pH 7.0 [8]. In our study, the deficient function of PMA1 resulted in a lower pH in the cytosol; whether V-ATPase activity can be stimulated by the observed lower intracellular pH in the absence of PMA1 function is unknown. 3,5-bisphosphate (PI(3,5)P_2_) plays a critical role in activating V-ATPase activity in response to salt stress [33], and the loss of Aly1 or Aly2 function results in increased accumulation of PI3P [32], which can be converted to PI(3,5)P_2_. These studies offer a clue that the loss of Aly1 or Aly2 function in cells lacking PMA1 activity might partially rescue the intracellular pH homeostasis by stimulating V-ATPase activity through the modulation of lipid composition.

## Figures and Tables

**Table 1:** Suppressor of *PMA1* in *Schizosaccharomyces pombe* (fission yeast) and the ortholog in *Saccharomyces cerevisiae* (budding yeast).

NO.	Ver4.0 Position	Systemic ID	Ortholog in Budding Yeast	Gene Description
1	V4-P02-12 (A12)	SPAC12G12.13c	YNL299W| YOL115W	poly(A) polymerase Cid14
2	V4-P02-54 (E6)	SPAC13G7.11	YBR185C	mitochondrial respiratory complex assembly protein (predicted)
3	V4-P04-33 (C9)	SPAC17G8.13c	YBL052C	histone acetyltransferase Mst2
4	V4-P04-46 (D10)	SPAC1805.01c	YAL017W| YOL045W	serine/threonine protein kinase Ppk6 (predicted)
5	V4-P06-75 (G3)	SPAC22H10.09	NONE	sequence orphan
6	V4-P07-62 (F2)	SPAC25B8.18	YKR049C	mitochondrial electron carrier (predicted)
7	V4-P12-04 (A4)	SPAC6G10.08	YDL066W|YLR174W|YNL009W	isocitrate dehydrogenase Idp1 (predicted)
8	V4-P13-47 (D11)	SPACUNK4.12c	YLR389C	metallopeptidase (predicted)
9	V4-P17-74 (G2)	SPBC18H10.06c	YKL018W	Set1C complex subunit Swd2.1
10	V4-P18-15 (B3)	SPBC19G7.04	NONE	HMG box protein
11	V4-P19-85 (H1)	SPBC2D10.04	YJL084C| YKR021W	arrestin Aly1 related, implicated in endocytosis
12	V4-P22-41 (D5)	SPBC609.02	YNL128W	phosphatidylinositol-3,4,5-trisphosphate3-phosphatasePtn1
13	V4-P25-06 (A6)	SPCC1235.09	YBR103W	Set3 complex subunit Hif2
14	V4-P26-63 (F3)	SPCC1906.02c	YGL110C	CUE domain protein Cue3 (predicted)
15	V4-P27-17 (B5)	SPCC31H12.05c	YER133W	serine/threonine protein phosphatase Sds21
16	V4-P28-81 (G9)	SPCC794.10	YHL012W| YKL035W	UTP-glucose-1-phosphate uridylyltransferase (predicted)
17	V4-P30-05 (A5)	SPAC186.02c	NONE	hydroxyacid dehydrogenase (predicted)
18	V4-P33-74 (G2)	SPBC29A3.21	NONE	sequence orphan
19	V4-P34-95 (H11)	SPBPB10D8.07c	YPL092W	membrane transporter (predicted)
20	V4-P35-39 (D3)	SPCC162.11c	YNR012W	uridine kinase/uracil phosphoribosyltransferase (predicted)
21	V4-P35-50 (E2)	SPCC16C4.17	NONE	meiotically upregulated gene Mug123

## Data Availability

The authors confirm that the data supporting the findings of this study are available within the article and its supplementary materials.

## Supplementary Materials

The following supporting information can be downloaded at https://www.mdpi.com/article/10.3390/cimb46010013/s1.
