# Supplementary material for: Identification of Aly1 and Aly2 as Modulators of Cytoplasmic pH in Saccharomyces cerevisiae
Source: Curr Issues Mol Biol. 2023 Dec 25;46(1):171–82. doi: 10.3390/cimb46010013 (PMC10814103; doi:10.3390/cimb46010013)
Supplement: Supplementary file 1 [file cimb-46-00013-s001.zip › cimb-2711493-supplementary.pdf]

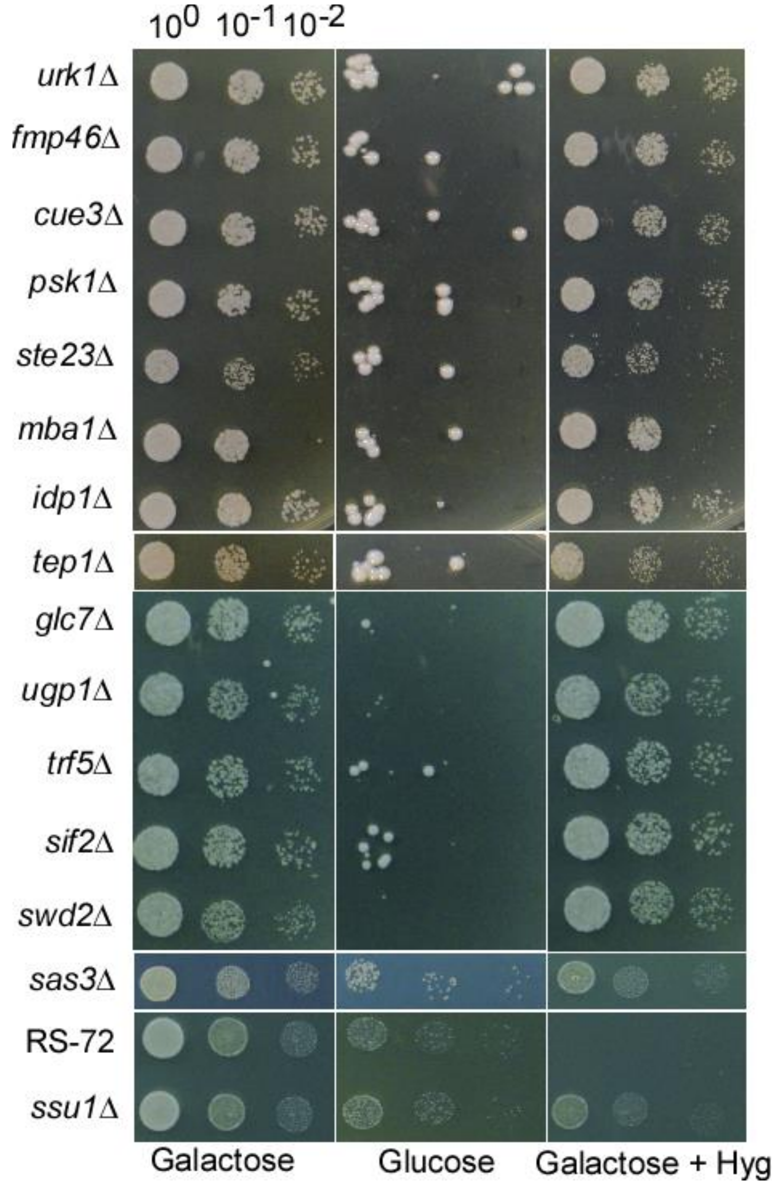

Figure S1. Other deletions (genes shown in Table 1) in the budding yeast strain RS-72 cannot rescue growth when a reduction in *Pma1* expression due to grow on the medium with glucose as the sole carbon source.

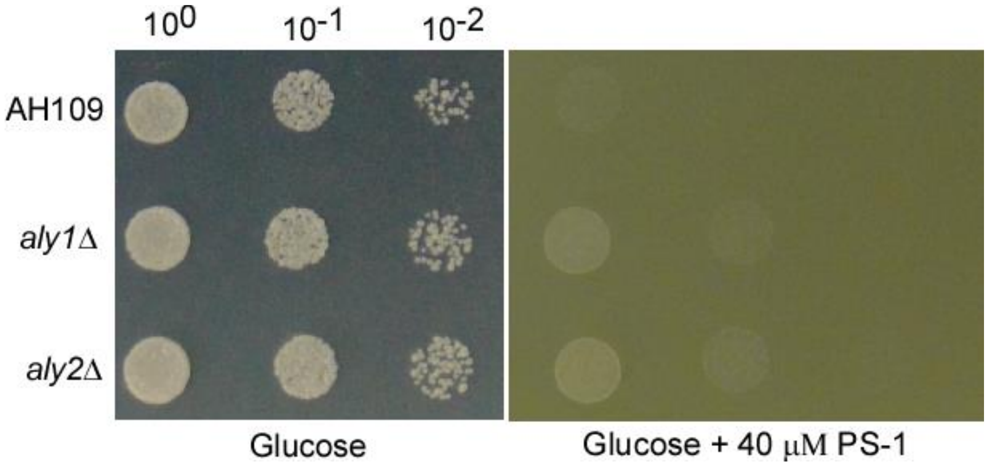

Figure S2. Deletion of *ALY1* or *ALY2* in AH109 strain show better resistance to PS-1 than AH109.

Table S1. Primers used in gene deletion assay.

Primer	Sequence (5′-3′)
5'-aly1-HygR-F	CTTGTTCCTCTATGACTACATGCTCCAATTCAATACAGAACGCGGCCGCCAGCTGAAGC
3'-aly1-HygR-R	CGGGAGACGAAAGAACTTCTTTTGGGAAGAAAATATTGGCAGTATAGCGACCAGCATTC
5'-aly2-HygR-F	CAGTCATTTTTCAAGATGCCCATGGACCAATCTATCTCAACGCGGCCGCCAGCTGAAGC
3'-aly2-HygR-R	GGAAAAGAAGCAGGATGTCCATCATAGCTCGGTAAAAGGCAGTATAGCGACCAGCATTC
5'-sas3-HygR-F	TTTGCAGATAATGTCATTAACAGCAAACGACGAATCGCCACGCGGCCGCCAGCTGAAGC
3'-sas3-HygR-R	GCTCCAAGTTTTGTAAATATTTTCAATCCTGTTCCAAGACAGTATAGCGACCAGCATTC
5'-swd2-HygR-F	GTCCTTTTAGCATAGCCAACAATGACCACCGTGTCCATCACGCGGCCGCCAGCTGAAGC
3'-swd2-HygR-R	ATGTATAATGGGAAACTATTGGCCGATGCAACGGTCGCGCAGTATAGCGACCAGCATTC
5'-cue3-HgR-F	GTTATCAAGGTACAACCGCGTTATTGAAATAAATGGGGGACGCGGCCGCCAGCTGAAGC
3'-cue3-HgR-R	TTTGGCTATCACGATTATGAACATAAGACGTTTTCCCTGCAGTATAGCGACCAGCATTC
5'-fmp46-HgR-F	ATGTCGTTTTGGAAAACATTGCAGAGACAGCCACGTACCACGCGGCCGCCAGCTGAAGC
3'-fmp46-HgR-R	ATGATGTACTTGTCAATGTCTGCGGGCTCATTTCCCACCCAGTATAGCGACCAGCATTC
5'-glc7-HgR-F	GGACTCACAACCAGTTGACGTTGATAATATCATCGATAGACGCGGCCGCCAGCTGAAGC
3'-glc7-HgR-R	CTTTCTACCCCCAGCTTGCCTTGGTAGACTTTTTTGGGCCAGTATAGCGACCAGCATTC
5'-mba1-HgR-F	GAGTGTATTAAGATCTACATGCCTTTTCTTCCCTCCAAGACGCGGCCGCCAGCTGAAGC
3'-mba1-HgR-R	TAGCTTGGAGGTAAACGATATATATCACCGTTAACTTTCCAGTATAGCGACCAGCATTC
5'-sif2-HgR-F	GAGTATAACAAGTGAAGAACTAAACTACTTGATATGGAGACGCGGCCGCCAGCTGAAGC
3'-sif2-HgR-R	CTACAACTGAACCTTCTTGAAGCGAGTATGCTACAGAAACAGTATAGCGACCAGCATTC
5'-ssu1-HgR-F	ATGGTTGCCAATTGGGTACTTGCTCTTACGAGGCAGTTTACGCGGCCGCCAGCTGAAGC
3'-ssu1-HgR-R	GCTAAACGCGTAAAATCTAGAGCCGAGTTTGATTCTTCCCAGTATAGCGACCAGCATTC
5'-ste23-HgR-F	ATGGGCGTATCTCTTTTAGCTTCTTCTTCTGCATTTGTAACGCGGCCGCCAGCTGAAGC
3'-ste23-HgR-R	GACGTCATCGGATAATAACAAAACAGGTTCATCTAATGGCAGTATAGCGACCAGCATTC
5'-tep1-HgR-F	ATGAGAGAGGAGGGGAGTGAATTAGAAATGGAAAAAGGCACGCGGCCGCCAGCTGAAGC
3'-tep1-HgR-R	TCCAATTTCAGTGCCTGAAATAATTTCAAGCCGTGAGTGCAGTATAGCGACCAGCATTC
5'-trf5-HgR-F	ATGACAAGGCTCAAAGCAAAATATTCACCTACAAAAGGTACGCGGCCGCCAGCTGAAGC
3'-trf5-HgR-R	GCTTCTGCTCACTCCAAAATCCGGAGGTGGAGATGGAATCAGTATAGCGACCAGCATTC
5'-ugp1-HgR-F	GTCCACTAAGAAGCACACCAAAACACATTCCACTTATGCACGCGGCCGCCAGCTGAAGC
3'-ugp1-HgR-R	TCAATGTTCCAAGATTTGCAAATTACCAGTAACGACAACCAGTATAGCGACCAGCATTC
5'-urk1-HgR-F	CCACCATGGACAACTCCATATATAATAGGTATAGGTGGTACGCGGCCGCCAGCTGAAGC
3'-urk1-HgR-R	AACCAAAATACTTTGAATCAAAAAATCTGGTCAATGCCCCAGTATAGCGACCAGCATTC
5'-idp1-HgR-F	TGAGTATGTTATCTAGAAGATTATTTTCCACCTCTCGCCACGCGGCCGCCAGCTGAAGC
3'-idp1-HgR-R	GTAGTCTTTTTTCAACGGCATCCAAAAATTCTTCTGTGGCAGTATAGCGACCAGCATTC
5'-psk1-HgR-F	ATGCCCTACATCGGTGCTTCCAACCTCTCAGAACATTCAACGCGGCCGCCAGCTGAAGC
3'-psk1-HgR-R	CTAATAAGCCGTTTTTCCTTTTTGCCCAAAACGATGCCCCAGTATAGCGACCAGCATTC
check-HygR-R	ATGGGGATCAGCAATCGCGC
check-aly1-F	GCTGCGTGGAAACAAGAGGC
check-aly2-F	CACCTTTAGATGTCTCGGCC
check-cue3-F	CTGATACAAGTGATCCGAGC
check-fmp46-F	CCTATTCTTCGCAGGAGATC

check-glc7-F	GTAGGTGCACTATTGGTTGC
check-idp1-F	GGAGCAATAGTCATGTCTGT
check-mba1-F	TCGCCAACGATGAAAAAGAG
check-psk1-F	TCCATGGCGGGTAATAAATA
check-sif2-F	GTTTCTGGCCTTCTTCAAAG
check-ssu1-F	GCAGTGTATCGTATAAGGCA
check-ste23-F	CCATTTCTTCATCGCGTTTC
check-tep1-F	AGATATTAGTGCGTGCTCCC
check-trf5-F	GACAACTTAAACTAGGAAGG
check-ugp1-F	GTCCTTATTACGCACGTTGC
check-urk1-F	TAGCAGCTATCCGTTATTAC
check-sas3-F	AGATGTTTCTACCTTACTCG
check-swd2-F	CCGCTATCATTGATGCGTCC
